# Supplementary material for: Teacher acceptability of physically active learning in UK secondary schools – a mixed methods study
Source: PLoS One. 2025 Aug 14;20(8):e0328376. doi: 10.1371/journal.pone.0328376 (PMC12352667; doi:10.1371/journal.pone.0328376)
Supplement: S1 File — (PDF) [file pone.0328376.s001.pdf]

Topic	Central Questions	Probes
**Current Practices**	*Can you describe how much students stay in their seats during your class typically?*	- Does the amount vary between year groups?<br>- How important do you think it is that students move around sometimes within lessons?<br>- Do you think classroom movement has any impact on concentration or behaviour?
	Do you have an example of when you have got your students up and moving around within your class?	- How did this go?<br>- What motivated you to do it?<br>- Was there anything that could have gone better?<br>- How enjoyable was it to deliver?<br>- How do you think your students felt about it?
**Physically Active Learning (PAL) Acceptability**	*What is your understanding of PAL?*	- (Provide a definition if needed)
	How do you feel about the idea of PAL?	- What are your thoughts after watching the Ted Talk video?<br>- Can you describe what do you like/don't like about PAL?<br>- How much do you think you would enjoy delivering PAL?<br>- Can you imagine integrating PAL into your teaching practice?<br>- How appropriate do you think it might be for your subject?<br>- Do you think PAL might work well for other subjects?<br>- How would you feel about collaborating with other subjects like PE or dance?
	Which type of PAL delivery most appeals to you?	- Why is that?<br>- How do you feel about PAL compared to movement breaks?<br>- Do you feel you could make adaptions for students who might find movement difficult?
	How do you imagine your students would react to PAL?	- How enthusiastically do you think they would participate?<br>- What would impact how they would react, in your opinion?<br>- How do you think PAL could help or hinder your students academically?<br>- Do you think it would benefit them in any other ways?
**Barriers and Facilitators**	*What would help you to be able to deliver PAL?*	- What would you need to feel confident or delivering PAL?<br>- How important is senior leadership team support?<br>- If they were on board, what would you need to take it forward?<br>- How much training do you imagine you would need and what would it look like?<br>- What type of support with lesson planning would you need?<br>- How important is it that PAL is school policy for you to be able to adopt it?
	What do you feel would stop you being able to deliver PAL?	- What concerns would you have?<br>- Do you imagine it would be a lot of extra work?<br>- Would you have enough time?<br>- How sufficient do you feel the space is, in or out of your classroom?
**Concluding Question**	*Following our discussion so far, is there a topic or idea that has come to mind in terms of how you could use PAL?*	- Is there any part of PAL that you could work with or implement?
