# Supplementary material for: Teacher acceptability of physically active learning in UK secondary schools – a mixed methods study
Source: PLoS One. 2025 Aug 14;20(8):e0328376. doi: 10.1371/journal.pone.0328376 (PMC12352667; doi:10.1371/journal.pone.0328376)
Supplement: S2 File — (PDF) [file pone.0328376.s002.pdf]

Name	Description	Files	References
Background Information	Relevant background information about each teacher and their school, for context	70	76
**Current Physical Activity**		0	0
Current class PA	Current levels and experiences of physical activity for secondary school pupils within classes	35	54
Examples of movement activities in class	Examples of movement activities that the secondary school teacher or their colleagues have previously delivered to their classes	19	35
Pupil's PA through the school day	Comments on the physical activity opportunities secondary school pupils have throughout the wider school day	10	12
**Perceived reactions/benefits for pupils**		0	0
Academic benefits	Perceived impact of physically active learning on academic skills and curriculum learning for secondary school pupils	27	54
Enjoyment of class PA	Perceived secondary school pupil enjoyment of physical activity within classes or physically active learning (experienced or hypothetical)	12	33
Focus and engagement	Perceived impact of physically active learning on the focus and engagement of secondary school pupils	28	42
Mental and physical health	Perceived impact of physically active learning on the mental and/or physical health of secondary school pupils	23	33
Perceived importance of variety for pupils	How secondary school teachers perceive variety within learning, including between lessons and within class activities, and how physically active learning might play a role in this	15	20
Other reactions & benefits for pupils	Other perceived reactions & benefits of physically active learning for secondary school pupils	14	37
**Individual factors for teachers**		0	0
General response to PAL	General response from secondary school teachers about physically active learning	66	106
Perceived importance of evidence	The extent to which secondary school teachers value evidence in introducing new initiatives such as physically active learning	8	26

Name	Description	Files	References
Teacher autonomy	Comments on how secondary school teacher autonomy might influence physically active learning implementation	6	11
Teacher confidence for PAL	How confident or competent teachers feel about delivering PAL	6	21
Teacher understanding of PAL	How secondary school teachers understand the concept of physically active learning	7	10
Teacher values of PA for pupils	The ways and extent to which teachers value physical activity for secondary school pupils	12	23
**Interpersonal factors**		0	0
Behaviour management	Perceived capability of teachers to manage class behaviour whilst delivering physically active learning	11	27
Behaviour of class	Perceptions of how physically active learning might reduce or increase behaviour issues of secondary school pupils, in classes or groups	26	47
Collaboration	Collaboration across subjects or with colleagues related to physically active learning, including cross-curricular collaboration	13	28
Differences - gender	Teachers' perceptions of the interaction between pupils' gender and classroom physical activity	19	26
Differences - learning styles and groups	Considerations and expectations of physically active learning implementation for pupils of differing learning styles or different groups	16	22
Differences - year groups or age	How secondary school teachers expect physically active learning to play out for pupils of different ages or year groups	17	29
Disabled or neurodivergent pupils	Expected adaptions, benefits and challenges of physically active learning for disabled or neurodivergent pupils	12	23
Inclusivity of PAL	Teacher perceptions of how and to what extent physically active learning might promote inclusivity; whether the approach can provide more equal opportunities for pupils from different backgrounds/abilities	17	24
Social factors for pupils	Perceived social factors for secondary school pupils related to physically active learning	15	30
**Institutional factors**		0	0
Differences - subjects	Teacher considerations of applying	13	28

Name	Description	Files	References
	physically active learning to different subjects in secondary schools.		
Physical environment in schools	Comments on the availability and use of facilities, space, and props within school, for current or hypothetical physically active learning	12	29
Teacher training	Comments on preferred teacher training for effective physically active learning implementation, as well as previous teacher training experiences	9	32
School environment - culture and norms		0	0
Differences - schools	Perceived differences between schools of varying types (e.g. state and independent), in how physically active learning might be implemented	10	23
Teacher buy-in	Perspectives on the buy-in of other secondary school teachers to adopt physically active learning	15	29
Time and academic pressures	Comments related to time and academic pressures on teachers in secondary schools	19	49
Other school culture or norms	Other factors related to school culture, values or norms	18	43
**PAL delivery**		0	0
Concerns about PAL delivery	Secondary school teacher concerns about the practicalities of physically active learning delivery	24	38
Implementation recommendations	Facilitators for implementing physically active learning in secondary schools, according to teachers	17	59
Outdoor PAL	Comments on and perceptions of outdoor physically active learning	7	32
PAL activity preferences	Physically active learning activity formats preferred by secondary school teachers	8	21
PAL vs movement breaks	Secondary school teachers' thoughts and preferences regarding physically active learning versus movement breaks (movement unrelated to academic learning)	10	14
Time needed to plan and deliver PAL	Comments on the time secondary school teachers expect physically active learning would take to plan and/or deliver	27	40
**Policy/education system factors**		0	0
Covid-19 Policy Factors	Perceived persisting impact of Covid-19	5	5

Name	Description	Files	References
	policies on pupil’s physical activity		
National policy and testing system	Teacher comments on how national policy and the wider educational testing system might interact with physically active learning	6	15
Perceptions of traditional learning	Teacher perceptions of traditional learning, how the current system compares, and what needs to change	6	15
School curriculum	Comments related to physically active learning and the school curriculum	8	11
School policy and SLT support	Factors relating to, and the perceived importance of, secondary school policy and senior leadership team support in physically active learning implementation	11	24
Other education system factors	Other perceived wider education system factors relevant to physically active learning	10	18
