# Supplementary material for: Teacher acceptability of physically active learning in UK secondary schools – a mixed methods study
Source: PLoS One. 2025 Aug 14;20(8):e0328376. doi: 10.1371/journal.pone.0328376 (PMC12352667; doi:10.1371/journal.pone.0328376)
Supplement: S3 File — (PDF) [file pone.0328376.s003.pdf]

Theme	Subtheme	Examples
**1) It's time for a change**	We need to move on from a Victorian view of education	*"…education definitely needs to change. You know. I think it is, it's quite sad in a way that um we're still approaching it in the way that they did, you know, over a hundred years ago. Um, society has moved on, and I think, I think we need to move on as well. […] we need to change the way we perceive, um, a good lesson or what learning should be about. And it's a shift in our thinking generally." (P6, female, languages)*
	National policies have exacerbated the neglect of movement in schools	"*With the new GCSEs being much more academically rigorous and time consuming, perhaps people have kind of forgotten about [movement in class] a bit, and that's why they're not necessarily using it […] [Since COVID-19], people just got used to students being in their seat, and teachers being at the front of the classroom […] we don't need to adhere to those strict regulations anymore. But people have got used to that again." (P1, female, languages)*
**2) PAL seems like common sense**	Some classroom movement already provided	"*For some concepts such as ratio it can be really helpful to have students play a role in the demonstration and move about themselves to visualise a problem." (P33, female, maths)*
	PAL is perceived to have important benefits	*"… everyone knows that unless kids are actually enjoying learning, they're not learning […] they're going to learn a lot more if they remember, "Oh my God! Today […] I learnt, you know, how to count to ten because we jumped up and down ten times…" (P3, female, special school)*<br><br>*"I like the idea that students do not have to be static in a classroom and can move around more, especially beneficial for those with ADHD and kinaesthetic learners." (P35, female, languages)*
**3) Is PAL delivery realistic in secondary schools?**	Examination and curriculum pressures	*"I could implement this idea easily, but its [sic] not a realistic expectation. I would need significantly more time to complete the curriculum with [sic] I wouldn't get." (P14, female, sciences)*
	Concerns about behaviour management and space	"*From experience there will always be students who use the time moving around assuming the cover of others and will move to hang round with friends. When there are 30-32 students moving round a space that doesn't accommodate for that many stood up it becomes difficult to monitor and help all students."* (P17, female, sciences)<br><br>*"…certain classrooms aren't really appropriate for moving around a lot. But then, that could be overcome in the fact that […] we have a massive yard which you can use, or why not go outside […] or collaborate with another subject?" (P6, female, languages)*
	PAL may be less suitable for girls and older pupils	*"…in particular Year 9 ,year 10 girls […] Not all. But some have that sort of opinion of […] they don't want to exercise or to show themselves moving around others, especially others of the different gender." (P7, male, PE)*
**4) Recommendations for implementation**	A consistent and collaborative, whole-school approach	*"School policy - needs to be integrated consistently across the school so that it is normalised and becomes routine" (P39, male, languages)*
	Evidence and resources are important	*"I think the biggest thing is resources and evidence. If it's going to be sold to staff and then to pupils and parents and guardians, there needs to be a clear, "This is the evidence that that shows that it is beneficial. And then, by the way for teachers, these are a big sample of resources of what you could do, or ideas or strategies that you could use within lesson, and how to do it."" (P7, male, PE)*
