# Supplementary material for: Teacher acceptability of physically active learning in UK secondary schools – a mixed methods study
Source: PLoS One. 2025 Aug 14;20(8):e0328376. doi: 10.1371/journal.pone.0328376 (PMC12352667; doi:10.1371/journal.pone.0328376)
Supplement: Attachment — Submitted filename: Letter - Teacher acceptability of pal; GMC.docx [file pone.0328376.s010.docx]

Dear authors,

Firstly, I would like to express my gratitude for the opportunity to collaborate on a research project of significant importance and relevance, particularly in light of the challenges posed by traditional teaching methods in the face of a technological surge that has the potential to distract many children and adolescents. The study emphasises that physical activity and more active learning methods are not yet accorded sufficient priority by many schools, with the focus remaining on curricular performance. It is not that the importance of curricular performance is diminished, but rather that a more active teaching method can contribute to the integral development of the individual.

Consequently, I would like to offer some observations on subjects that could benefit from further elaboration.

On line 153, page 7, the following assertion is made: As this exploratory study sought to establish a foundation for future research, the recruitment target was set at 75-150 survey respondents, with a subsample of 7-10 interviewees, based on the findings of previous similar studies [38, 50, 51, 52, 53, 54]. However, the text does not specify the number of participating schools or if a minimum number of schools was required for recruitment.

It is to be posited herewith that, in the event of 25 teachers from each school participating, a total of 75 individuals would be required. Conversely, should only 8 teachers from 10 schools participate, the resultant sample would already comprise 80 individuals. The issue under discussion is that of the representativeness of the schools. It is posited that the inclusion of a greater number of teachers from a given unit could give rise to problems.

It is acknowledged that further information can be found in other sources. The integration of a sentence could facilitate comprehension.

Despite the implementation of a multifaceted sampling strategy, encompassing purposive, convenience, and snowball sampling techniques, this approach may potentially compromise the representativeness of the participant cohort. This particular recruitment strategy tends to appeal to teachers who have already demonstrated a high level of engagement or interest in the domain of PAL (Physical Activity in Learning), thereby introducing a form of self-selection bias. This may compromise the generalisability of the findings. Despite the recognition of such limitations in previous literature, it is imperative to articulate the notion of self-selection bias.

Finally, it is imperative to emphasise the absence of validation for the employed measures, a subject that demands further attention. It is important to note that some of the questions in the questionnaire were created by the researcher himself. These were based on previous literature, but without undergoing a formal psychometric validation process. Moreover, the preliminary study was confined to a sample size of four participants, comprising one teacher and three peers. While this approach offers face validity, there is no guarantee that the items accurately capture the proposed constructs, such as acceptance, motivation, or perception of PAL. The adaptation of existing scales (for example, the Work Tasks Motivation Scale) involves changes in structure and items without revalidation, which may affect reliability and construct validity.
